# Potent Anti-Inflammatory Activity of Ursolic Acid, a Triterpenoid Antioxidant, Is Mediated through Suppression of NF-κB, AP-1 and NF-AT

**DOI:** 10.1371/journal.pone.0031318

**Published:** 2012-02-20

**Authors:** Rahul Checker, Santosh K. Sandur, Deepak Sharma, Raghavendra S. Patwardhan, S. Jayakumar, Vineet Kohli, Gautam Sethi, Bharat B. Aggarwal, Krishna B. Sainis

**Affiliations:** 1 Radiation Biology and Health Sciences Division, Bio-Medical Group, Bhabha Atomic Research Centre, Mumbai, India; 2 Medical Division, Bio-Medical Group, Bhabha Atomic Research Centre, Mumbai, India; 3 Department of Pharmacology, Yong Loo Lin School of Medicine, National University of Singapore, Singapore, Singapore; 4 Cytokine Research Laboratory, Department of Experimental Therapeutics, The University of Texas M.D. Anderson Cancer Center, Houston, Texas, United States of America; Institut Jacques Monod, France

## Abstract

**Background:**

Ursolic acid (UA), a pentacyclic triterpenoid carboxylic acid, is the major component of many plants including apples, basil, cranberries, peppermint, rosemary, oregano and prunes and has been reported to possess antioxidant and anti-tumor properties. These properties of UA have been attributed to its ability to suppress NF-κB (nuclear factor kappa B) activation. Since NF-κB, in co-ordination with NF-AT (nuclear factor of activated T cells) and AP-1(activator protein-1), is known to regulate inflammatory genes, we hypothesized that UA might exhibit potent anti-inflammatory effects.

**Methodology/Principal Findings:**

The anti-inflammatory effects of UA were assessed in activated T cells, B cells and macrophages. Effects of UA on ERK, JNK, NF-κB, AP-1 and NF-AT were studied to elucidate its mechanism of action. *In vivo* efficacy of UA was studied using mouse model of graft-versus-host disease. UA inhibited activation, proliferation and cytokine secretion in T cells, B cells and macrophages. UA inhibited mitogen-induced up-regulation of activation markers and co-stimulatory molecules in T and B cells. It inhibited mitogen-induced phosphorylation of ERK and JNK and suppressed the activation of immunoregulatory transcription factors NF-κB, NF-AT and AP-1 in lymphocytes. Treatment of cells with UA prior to allogenic transplantation significantly delayed induction of acute graft-versus-host disease in mice and also significantly reduced the serum levels of pro-inflammatory cytokines IL-6 and IFN-γ. UA treatment inhibited T cell activation even when added post-mitogenic stimulation demonstrating its therapeutic utility as an anti-inflammatory agent.

**Conclusions/Significance:**

The present study describes the detailed mechanism of anti-inflammatory activity of UA. Further, UA may find application in the treatment of inflammatory disorders.

## Introduction

Inflammation involves the activation and recruitment of phagocytes (macrophages, neutrophils), NK cells, complement system and secretion of cytokines like IL-1β, IL-6, TNF-α by activated cells which are essential for the host defence system. The chronic inflammation that persists even after elimination of pathogen(s) has been associated with several diseases such as cancer [1], [2], neoplasms, inflammatory bowel disease, ulcerative colitis [3], [4], atherosclerosis, rheumatoid arthritis [5], asthma and Alzheimer's disease [6]. The damaging responses resulting from chronic inflammation can be controlled by altering the molecular mediators of an inflammatory response. The key targets are proinflammatory cytokines and cytokine receptors (tumor necrosis factor (TNF-α) and TNF-RII, IL-12, IL-6, interferon-γ), enzymes (COX-2, inosine monophosphate dehydrogenase), cell surface molecules required for intercellular interactions and leukocyte activation. Dysregulation of these cytokines and enzymes may contribute to the pathogenesis of many chronic inflammatory diseases [7], [8]. Transcription factors (NF-κB, NF-AT and AP1) along with mitogen-activated protein kinases (ERK, JNK, and p38) are known to regulate these inflammatory cytokines and enzymes and are being targeted by several investigators to ameliorate chronic inflammation [9], [10], [11], [12], [13], [14], [15]. There are several reports showing the involvement of reactive oxygen species (ROS) and glutathione (GSH) in modulating these immunologically important kinases and transcription factors and resulting in altered immune responses [16], [17]. The treatment approaches for these inflammatory disorders are addressed by administration of steroids and non-steroidal anti-inflammatory drugs (NSAID) like azathioprine. All of these drugs are accompanied by an array of side effects [18], [19], [20], [21] restricting their continuous usage and complicating treatment modalities. Thus there is a need to identify safe and non-toxic anti-inflammatory drugs from plant sources that can be used for the treatment of inflammatory disorders.

Ursolic acid, (UA; 3b-hydroxy-12-urs-12-en-28-oic acid), a natural pentacyclic triterpenoid carboxylic acid is present in a wide variety of plants, including apples, basil, bilberries, cranberries, peppermint, rosemary, oregano [22]. Several biochemical and pharmacological effects of UA such as anti-inflammatory, antioxidant, anti-proliferative, anti-cancer, anti-mutagenic, anti-atherosclerotic, anti-hypertensive, anti-leukemic and antiviral properties are reported in a number of experimental systems [23], [24]. UA exhibited anti-inflammatory effects in RAW264.7 cells (Mouse monocyte macrophage cell line) by attenuating inducible nitric oxide synthase and cycloxygenase-2 expression [25], [26]. The anti-proliferative, anti-tumor and anti-leukemic properties have been shown to be mediated via suppression of NF-κB activation and inhibiting the expression of NF-κB regulated genes like lipoxygenase, COX-2, MMP-9, and iNOS [27], [28], [29], [30].

It is well known that activation of NF-κB, MAPKs, AP-1 and NF-AT following major histocompatible complex-T cell receptor (MHC-TCR) interaction is vital for the antigen induced lymphocyte proliferation, cytokine secretion and survival [31]. In resting T cells, NF-κB is sequestered into an inactive state by the cytoplasmic inhibitor of NF-κB (IκBα). T cell activation through TCR leads to the rapid activation of the IκB kinases (IKKs) via protein kinase C and results in phosphorylation and subsequent degradation of IκB proteins which allows nuclear translocation of NF-κB [32]. Since dysregulation of NF-κB function is associated with inflammation, any molecule that interferes with NF-κB activation is a potential candidate for therapeutic strategy in the treatment of inflammatory diseases. The present study was aimed to investigate anti-inflammatory properties of UA in murine lymphocytes. The molecular mechanism of action of UA for the observed anti-inflammatory activity was also studied.

## Results

### UA inhibited Con A and anti-CD3/CD28 mAb induced proliferation of lymphocytes in vitro

**Figure 1:**
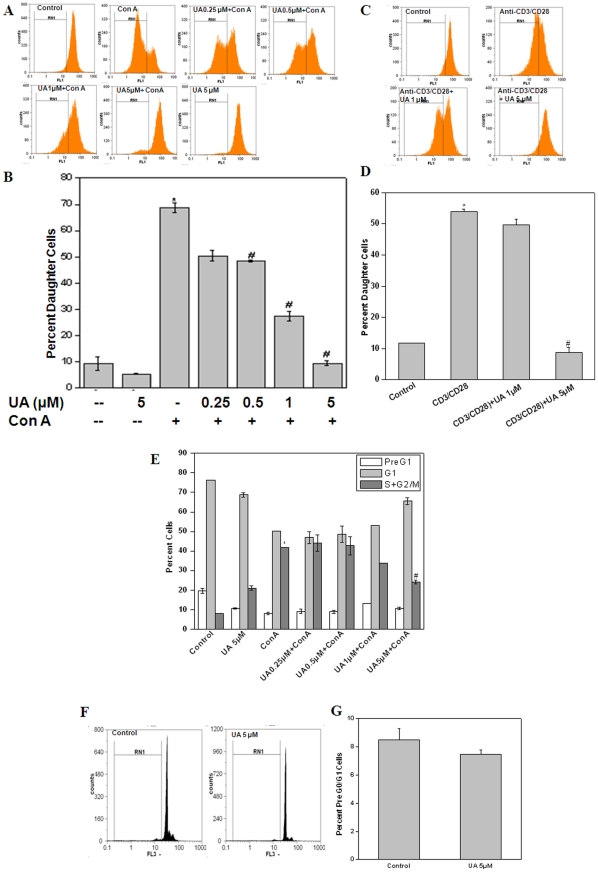
Ursolic acid inhibits lymphocyte proliferation in vitro. (A) For cell proliferation analysis CFSE labelled lymphocytes were treated with UA (0.25–5 µM, 4 h) and stimulated with the Con A (10 µg/ml) at 37°C for 72 h. Twenty thousand cells were acquired in a flowcytometer. Vehicle treated cells served as control. Percent daughter cells were calculated from decrease in mean fluorescence intensity. (B) Each bar represents percentage of daughter cells in each treatment group. (C & D) UA inhibits anti-CD3 induced T cell proliferation. CFSE labelled lymphocytes were treated with UA (1 & 5 µM, 4 h) and stimulated with coated anti-CD3mAb (1 µg/ml) and soluble anti CD28mAb (1 µg/ml) at 37°C for 72 h. Percent daughter cells were estimated by CFSE dye dilution. Representative histogram and corresponding bar diagram are shown in Fig. 1C and D respectively. (E) UA inhibits Con A induced cell cycle progression: Lymphocytes were treated with UA (0.25–5 µM, 4 h) and stimulated with the Con A (5 µg/ml) at 37°C for 72 h. The cells were stained with propidium iodide and twenty thousand cells were acquired in a flowcytometer. The hollow bars represent percentage of cells containing less than 2n DNA (sub-G1/apoptotic cells), light gray bars show cells containing 2n DNA (in G1 phase) and the dark gray bars indicate the cells containing more than 2n DNA (in S+G2/M phase). (F) Evaluation of the potential cytotoxicity of UA to lymphocytes. Lymphocytes were cultured with UA (5 µM) for 24 h and cytotoxicity was measured by PI staining. Vehicle treated cells served as control. Percentage apoptosis (pre-G1 peak) in lymphocytes was estimated and is shown in the histograms. (G) Each bar represents percentage of apoptotic in each treatment group. Data points represent mean±S.E.M. from three replicates and three such experiments were carried out. *p<0.01, as compared to vehicle treated cells and #p<0.01, as compared to Con A or antiCD3/CD28 stimulated cells.

The potential immunomodulatory effects of UA were studied by stimulating murine splenic lymphocytes with polyclonal T cell mitogen Con A or with plate bound anti-CD3 plus soluble anti-CD28 mAb in the presence or absence of UA. Lymphocyte proliferation induced by Con A or anti-CD3/CD28 mAb was assessed by CFSE dye dilution using a flowcytometer. As shown in figure 1A–D, UA inhibited Con A induced lymphocyte proliferation in a dose dependent manner in vitro. UA at 5 µM completely inhibited both Con A and anti-CD3/CD28 mAb induced lymphocyte proliferation (Fig. 1B & D). This inhibition of proliferation may be due to inhibition of entry of cells into S phase of the cell cycle as evinced from cell cycle analysis (Fig. 1E). The fraction of cells in S+G2/M phase of cell cycle in UA treated lymphocytes stimulated with Con A was significantly lower than that in lymphocytes stimulated with Con A alone (Fig. 1E). There was a concomitant increase in the percentage of cells in G1 phase of cell cycle in UA treated lymphocytes stimulated with Con A than that in lymphocytes stimulated with Con A alone indicating that UA induced G1 phase arrest in activated T cells (Fig. 1E). This inhibition of mitogen and anti-CD3/CD28 mAb induced T cell proliferation was not due to induction of cell death as this inhibitory concentration of UA was found to be non-toxic to lymphocytes when assessed by propidium iodide (PI) staining (Fig. 1F&G).

### UA inhibited proliferation of CD4+ T cells, CD8+ T cells and B cells

**Figure 2:**
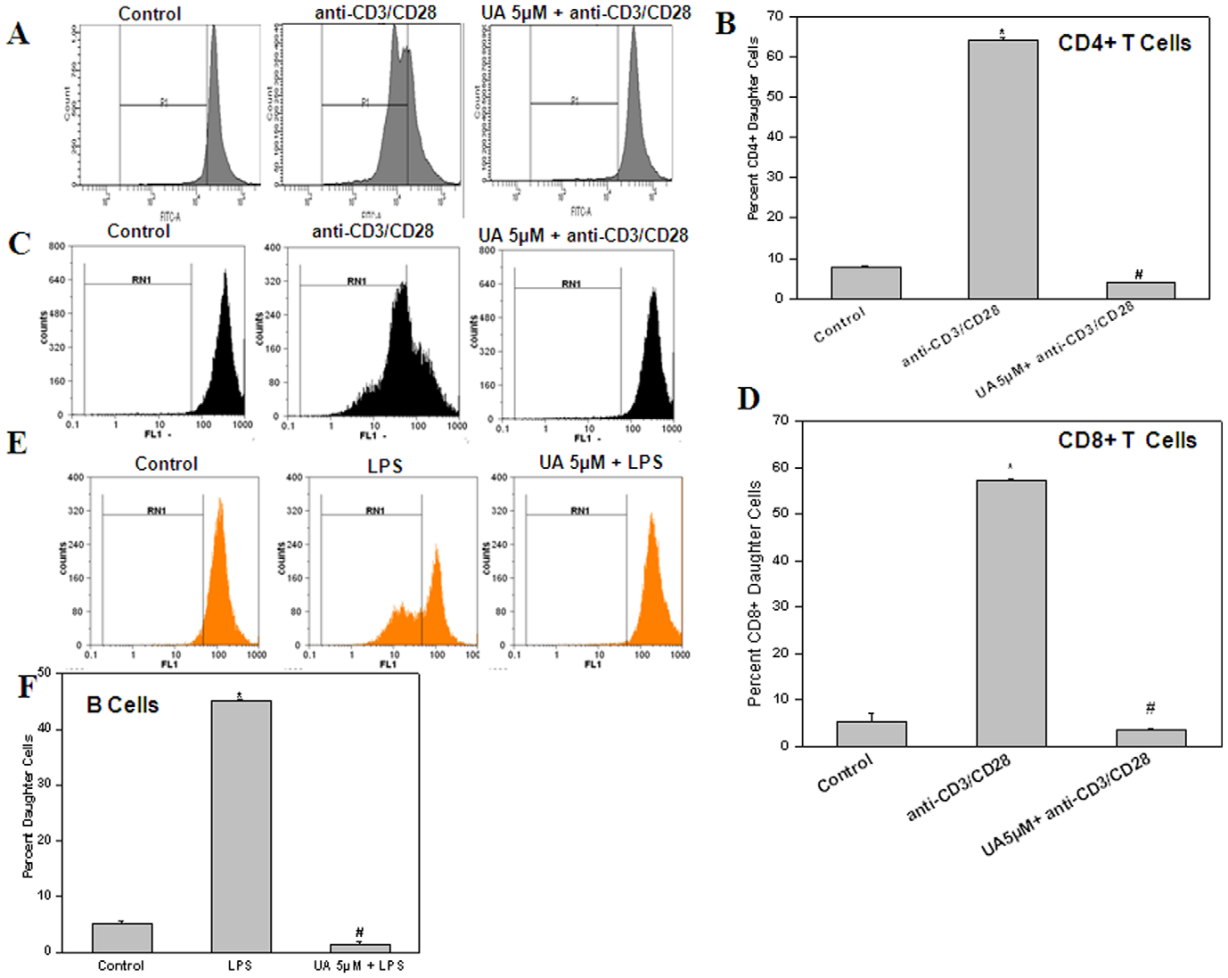
Ursolic acid inhibits mitogen induced proliferation of CD4+ T cells, CD8+ T cells and B cell. CFSE labelled purified CD4+T cells (A) or CD8+Tcells (C) were pre-treated with ursolic acid (5 µM, 4 h) before stimulation with coated anti-CD3mAb (1 µg/ml) and soluble anti CD28mAb (1 µg/ml) for 72 h at 37°C. Percent daughter cells were calculated from decrease in mean fluorescence intensity. (B&D) Each bar represents percentage of daughter cells in different treatment groups. (E,F) UA inhibits LPS induced proliferation of B cells. CFSE labeled lymphocytes were treated with UA (5 µM, 4 h) and stimulated with the LPS (50 µg/ml) at 37°C for 72 h. Vehicle treated cells served as control. Percent daughter cells were calculated from decrease in mean fluorescence intensity. Each bar represents mean±S.E.M. from three replicates and two such independent experiments were carried out. *p<0.01, as compared to vehicle treated cells and #p<0.01, as compared to Con A or LPS or anti-CD3/anti-CD28 stimulated cells.

T cells (CD4+ and CD8+) and B cells are the two major cell types involved in the adaptive immune response following pathogenic invasion. We studied whether UA acts on both these cell types or is specific to a particular lineage of lymphocytes. As shown in Fig. 2A–F, UA inhibited anti-CD3/CD28 mAb induced proliferation of CD4+ T cells (Fig. 2A&B), CD8+ T cells (Fig. 2C&D) and LPS stimulated proliferation of B cells (Fig. 2E&F).

### UA inhibited Con A, anti-CD3/CD28 mAb and LPS induced cytokine secretion by lymphocytes, CD4+ T cells and macrophages in vitro

**Figure 3:**
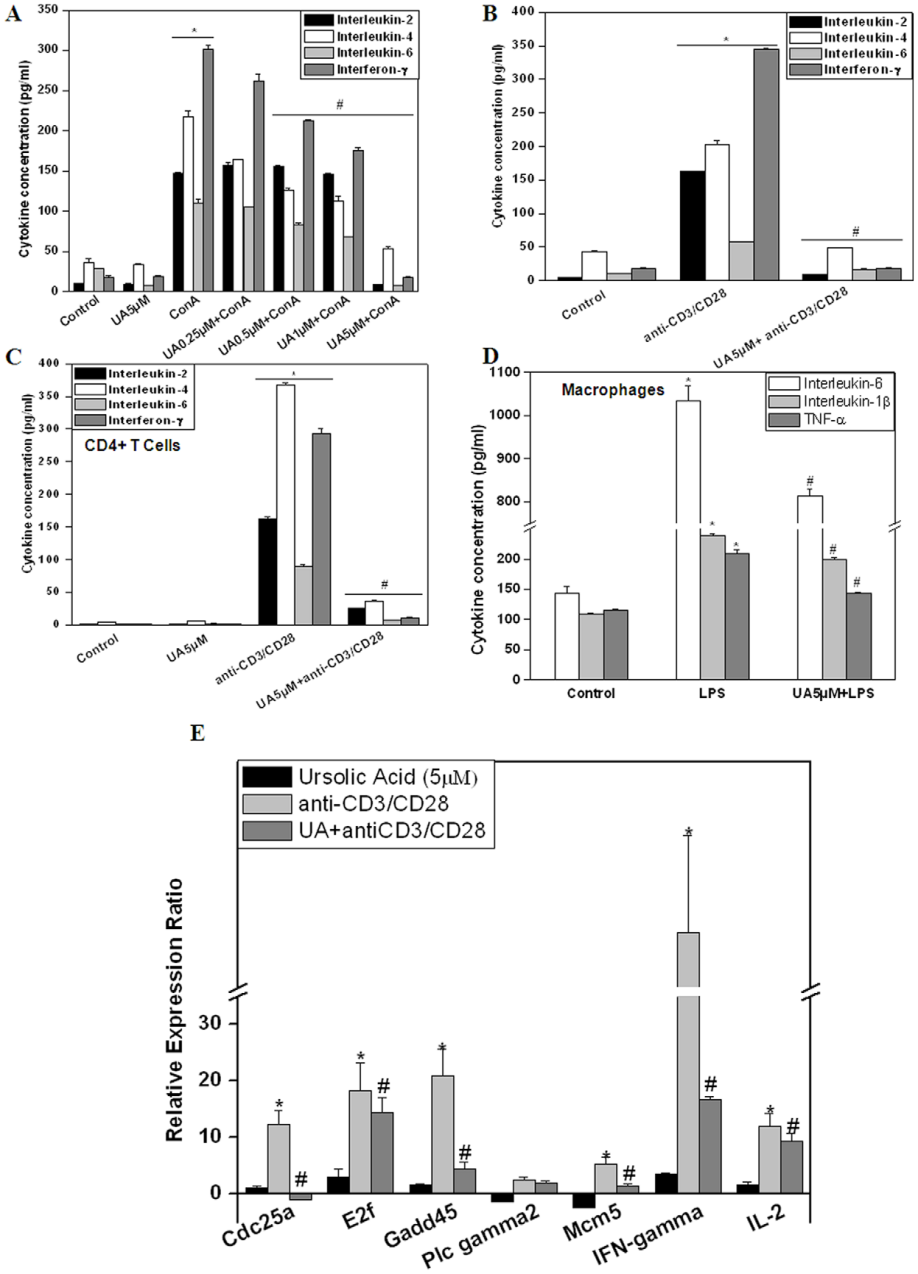
Ursolic acid inhibits cytokine secretion by T cells and macrophages. Lymphocytes were pre-treated with different concentrations of UA for 4 h before stimulation with Con A (5 µg/ml) (A) or anti-CD3/anti-CD28 mAb (B) for 24 h at 37°C. Vehicle treated cells served as control. The concentration of cytokines in the culture supernatant was estimated using ELISA. (C) UA inhibits anti-CD3/anti-CD28 induced cytokine secretion by CD4+T cells. Purified CD4+T cells were pre-treated with different concentrations of UA for 4 h before stimulation with anti-CD3/anti-CD28 mAb for 24 h at 37°C. The concentration of cytokines in the supernatant was estimated using ELISA. (D) UA inhibits LPS induced cytokine production from macrophage. Splenic adherent macrophage were pre-treated with UA (5 µM, 4 h) and then stimulated with LPS (50 µg/ml) for 24 or 48 h. The concentration of cytokines in the supernatant was estimated using ELISA. Each bar represents mean±S.E.M. from three replicates and two such independent experiments were carried out. *p<0.01, as compared to vehicle treated cells and #p<0.01, as compared to Con A or LPS or anti-CD3/anti-CD28 stimulated cells. (E) Quantative real time RT-PCR analysis of differential genes expression in CD4+ T cells activated with antiCD3/CD28 in the presence or absence of Ursolic acid. Purified CD4+T cells were pre-treated with UA for 4 h before stimulation with anti-CD3/anti-CD28 mAb for 24 h at 37°C. The relative expression ratio was calculated and plotted as mean ± SEM. *p<0.01, as compared to vehicle treated cells and #p<0.01, as compared to anti-CD3/anti-CD28 stimulated cells.

Fig. 3 shows the secretion of IL-2, IL-4, IL-6 and IFN-γ cytokines by lymphocytes stimulated with Con A or anti-CD3/CD28 mAb in the presence or absence of UA. Lymphocytes stimulated with Con A or anti-CD3/CD28 mAb produced significantly higher levels of IL-2, IL-4, IL-6 and IFN-γ cytokines (Fig. 3A & B). Treatment of cells with UA (5 µM) completely inhibited both Con A and anti-CD3/CD28 mAb induced secretion of IL-2, IL-4, IL-6 and IFN-γ cytokines (Fig. 3A & B). These results were in agreement with the earlier results showing complete inhibition of Con A and anti-CD3/CD28 mAb induced proliferation of lymphocytes by UA at 5 µM (Fig. 1B&D). Similar anti-inflammatory effects of UA were observed on CD4+ T cells when they were stimulated with anti-CD3/CD28 mAb in the presence of UA. Treatment of purified CD4+ T cells with UA prior to stimulation with anti-CD3/CD28 mAb led to complete the inhibition of secretion of IL-2, IL-4, IL-6 and IFN-γ (Fig. 3C). It was also observed that treatment of splenic adherent macrophages with UA prior to stimulation with LPS completely inhibited the secretion of IL-6, IL-1β and TNF-α cytokine (Fig. 3D).

### Inhibition of gene expression in activated CD4+ T cells by ursolic acid

Quantitative real time RT-PCR for 6 genes that are known to be involved in T cell activation (IL-2), B cell activation (phospholipase C gamma 2 (Plcg2)), cell division/cycle (cell division cycle 25 homolog A (Cdc25a), E2F, growth arrest and DNA damage 45 gamma (Gadd45g) and minichromosome maintenance complex component 7 (Mcm7)) and functioning (IFN-g) was carried out. On activation with CD3/CD28 mAb the expression of all these genes was elevated in comparison to control (Fig. 3E). But treatment of cells with UA prior to activation with antiCD3/CD28 resulted in significant suppression of mRNA levels of these genes except for Plcg2 (Fig. 3E).

### UA inhibited up-regulation of activation markers and co-stimulatory molecules on both T and B cells

**Figure 4:**
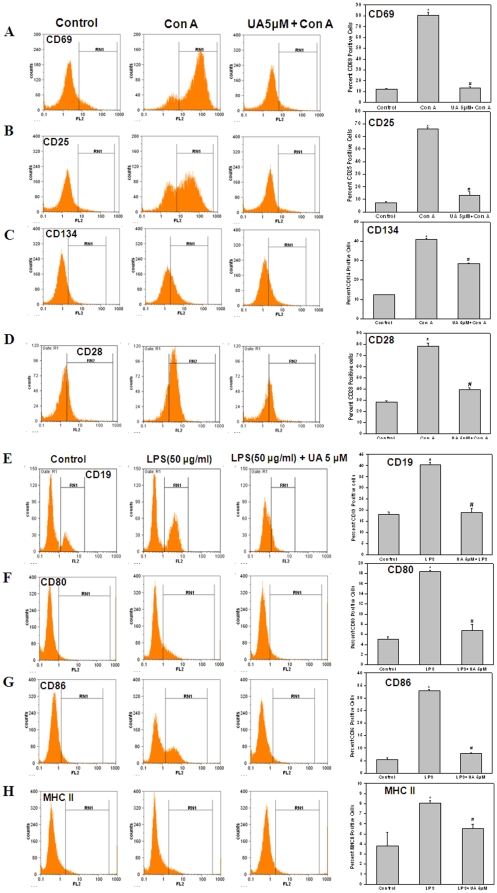
Ursolic acid suppresses inducible expression of T cell and B cell activation markers and co-stimulatory molecules. Lymphocytes were treated with UA (5 µM, 4 h) and then stimulated with Con A (10 µg/ml) or LPS (50 µg/ml) for 24 h (A–B) or 48 h(C–H) at 37°C. In each group, 1×10^6^ cells were stained with PE conjugated anti-CD69 (A) or anti-CD25 (B) or anti-CD134 (C) or anti-CD28 (D) or anti-CD19 (E) or anti-CD80 (F) or anti-CD86 (G) or anti-I-A (H) mAbs. Representative flowcytometric histogram and corresponding bar diagram are shown. Data points represent mean±S.E.M. from three replicates and two such independent experiments were carried out. *p<0.01, as compared to vehicle treated cells and #p<0.01, as compared to Con A or LPS stimulated cells.

Optimum T cell activation requires signaling through both T cell receptor as well as through co-stimulatory proteins. Activation of T cells only through TCR in the absence of a co-stimulatory signals leads to T cell anergy. Also, T cell upon activation up-regulate certain cell surface proteins which are necessary for complete T cell activation and effector functions. The effect of UA on T cell activation markers and co-stimulatory molecules was studied to determine whether UA inhibits T cell activation and induces T cell anergy. Fig. 4 shows the expression of early and late T cell activation markers CD69 (Fig. 4A), CD25 (IL-2R-α, Fig. 4B) and CD134 (Fig. 4C) and co-stimulatory molecule CD28 (Fig. 4D) in lymphocytes treated with UA (5 µM, 4 h) and stimulated with Con A (5 µg/ml). Mitogen activated cells showed significantly higher expression of CD69, CD25, CD134 and CD28 as compared to that in control cells (Fig. 4A–D). Treatment of lymphocytes with UA prior to Con A stimulation completely inhibited the Con A induced upregulation of CD69, CD134, CD25 and CD28 expression on T cells (Fig. 4A–D). Interaction of antigen bound MHC and CD80 (B7.1) and CD86 (B7.2) on antigen presenting cells with TCR and CD28 on T cells respectively provide the two signals required for complete T cell activation. Lack of either MHC-TCR or CD28-CD80 interaction impairs the immune response. We studied effects of UA on LPS induced upregulation of CD19, CD80, CD86 and MHC II on activated B cells. As shown in Fig. 4E-H, treatment of cells with UA prior to LPS stimulation inhibited the upregulation of CD19, CD80, CD86 and MHC II on activated leukocytes.

### Modulation of intracellular redox status by UA

**Figure 5:**
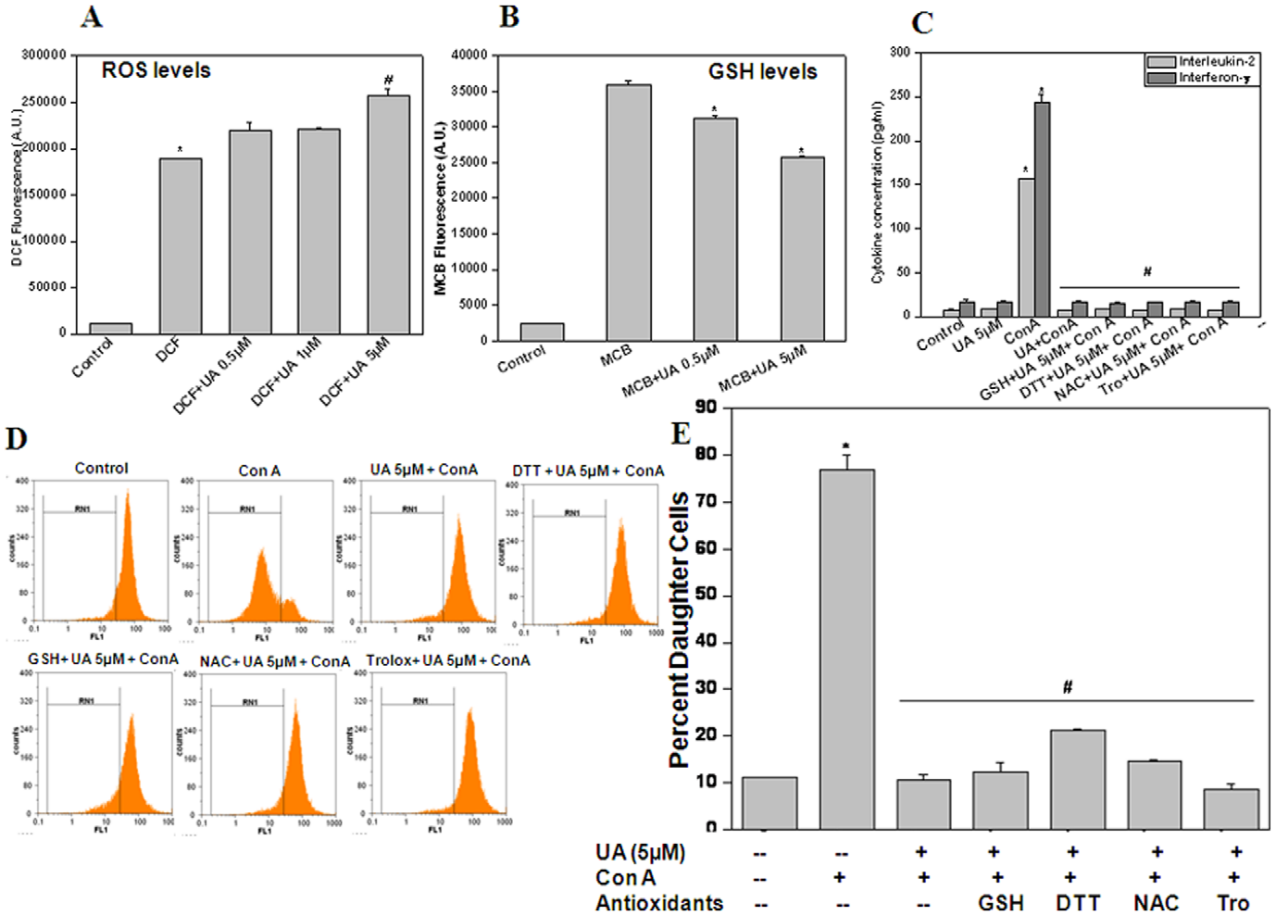
Ursolic acid Modulates cellular ROS levels. (A) UA increased ROS levels in lymphocytes. Lymphocytes were stained with DCF-DA (20 µM, 30 min at 37°C), treated with UA (0.5–5 µM) for 1 h and fluorescence emission was measured at 535 nm. *p<0.01, as compared to DCF treated cells (B) Effect of UA on intracellular GSH levels. Lymphocytes were treated with UA for 4 h at 37°C and stained with MCB (final concentration 40 µM) for 30 min. Fluorescence emission was measured at 490 nm following excitation at 394 nm. *p<0.01, as compared to MCB treated cells (C) Immunosuppressive effects of UA were independent of cellular redox status. Lymphocytes were incubated with different antioxidants (GSH or NAC or DTT or trolox) for 2 h. These cells were then stimulated with Con A in presence or absence of UA for 24 h at 37°C .The concentration of IL-2 and IFN-γ in the culture supernatant was estimated by ELISA. (D&E) Lymphocytes were stained with CFSE and were incubated with different antioxidants (GSH 10 mM or NAC 10 mM or DTT 100 µM or trolox 100 µM) for 2 h. The cells were then stimulated with Con A in presence or absence of UA for 72 h at 37°C. Cell proliferation was measured from CFSE dye dilution using a flowcytometer. Representative flowcytometric histograms and corresponding bar diagram are shown (Fig. 5D&E). Each bar shows mean±S.E.M from three replicates and two such independent experiments were carried out. *p<0.01, as compared to vehicle treated cells and #p<0.01, as compared to Con A stimulated cells.

Intracellular ROS and GSH levels are known to pay an important role in immune responses and several molecules have been shown to exhibit their immunomodulatory activity via a redox dependent manner [33]. Hence, we examined whether UA also acts in a similar manner via modulation of cellular redox status. Treatment of lymphocytes with UA significantly increased the DCF (Dichlorodihydrofluorescein) (Fig. 5A) fluorescence at 5 µM. To ascertain whether the observed increase in intracellular ROS is accompanied with a concomitant decrease in GSH levels we checked the levels of intracellular GSH in lymphocytes following UA treatment. We observed that 4 h after UA treatment there was a significant decrease in the levels of GSH in lymphocytes (Fig. 5B). To determine the role of redox in the observed anti-inflammatory effects of UA we checked whether antioxidants could abrogate the suppressive effects of UA. Fig. 5C–E shows the effect of thiol and non thiol antioxidants on the suppressive effect of UA on Con A induced proliferation and cytokine secretion of lymphocytes. The suppression of Con A induced lymphocyte proliferation and cytokine secretion by UA could not be abrogated by thiol (glutathione (GSH), N-acetylcysteine (NAC) and dithiothreitol (DTT)) or non-thiol antioxidant (trolox) suggesting that the effects of UA are independent of cellular redox status (Fig. 5C–E).

### UA suppressed mitogen induced MAPK, NF-κB, NF-AT and AP-1 activation in lymphocytes

**Figure 6:**
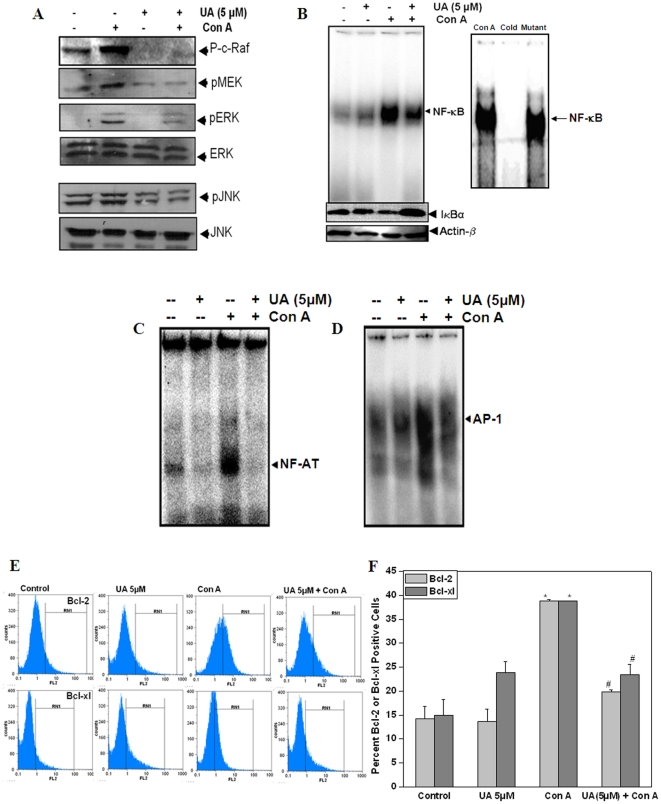
Inhibition of proliferation/survival associated signaling molecules by ursolic acid in activated T cells. Lymphocytes were incubated with UA (5 µM, 4 h) and stimulated with Con A (5 µg/ml) for 1 h. Whole cell lysates were prepared, fractionated on 10% SDS–PAGE, and electrotransferred to nitrocellulose membrane. Western blot analysis was performed using different antibodies specific for (A) p-c-Raf, p-MEK, p-ERK, ERK, p-JNK and JNK (B) IκBα and β-Actin (loading control). (C–E) Lymphocytes were incubated with 5 µM UA for 4 h and then stimulated with Con A (5 µg/ml) for 1 h. Nuclear extracts were prepared and analysed for NF-κB (B), NF-AT (C) and AP-1 (D) activation by EMSA. (E&F), UA inhibits NF-κB dependent gene products in activated lymphocytes. Lymphocytes were incubated with UA (5 µM, 4 h) and were stimulated with Con A (5 µg/ml) for 24 h, harvested, fixed, permeablized and stained with PE labelled Bcl-2 antibody or Bcl-xl antibody. Representative flowcytometric histograms (E) and the corresponding bar diagram (F) are shown. Each bar shows mean±S.E.M from three replicates and two such independent experiments were carried out. *p<0.01, as compared to vehicle treated cells and #p<0.01, as compared to Con A stimulated cells.

It is well known that following MHC-TCR engagement, a large number of proteins including MAPkinases, NFκB, AP1 and NF-AT are activated which co-ordinate with each other resulting in an immune response. To study the molecular mechanism of action of UA, we studied its ability to modulate signaling events that are involved in T cell activation. Fig. 6, shows the effect of UA on Con A induced MAPKinases (MEK, ERK and JNK), NF-κB, AP-1 and NF-AT activation in lymphocytes. Treatment of lymphocytes with UA inhibited Con A induced ERK and JNK phosphorylation. The observed inhibition of ERK phosphorylation may be due to the inhibition of mitogen induced phosphorylation of c-raf and MEK which are upstream of ERK and are responsible for ERK phosphorylation upon activation (Fig. 6A). Lymphocytes treated with Con A (5 µg/ml) for 1 h showed degradation of IκB-α in the cytosolic fraction and activation of NF-κB, NFAT and AP-1 in the nuclear fraction as compared to that in vehicle treated control cells (Fig. 6B–D). However, cells treated with UA (5 µM) and then stimulated with Con A (5 µg/ml, 1 h) did not show IκB-α degradation (Fig. 6B). UA suppressed Con A mediated activation of all three important immunoregulatory transcription factors NF-κB, NFAT and AP-1 (Fig. 6B–D). The addition of excess unlabeled NF-κB (cold oligonucleotide, 100-fold) caused a complete disappearance of the band, whereas mutated oligonucleotide had no effect on DNA binding suggesting that the band belongs to NF-κB(Fig. 6B). Fig. 6E&F shows the effect of UA on Con A induced upregulation of NF-κB dependent genes in lymphocytes. Stimulation of lymphocytes with Con A (5 µg/ml) for 24 h resulted in significant upregulation of Bcl-2 and Bcl-xl protein levels (Fig. 6E&F). This increase in the levels of NF-κB dependent proteins (Bcl-2 and Bcl-xl) in Con A activated lymphocytes was inhibited by treatment with UA (Fig. 6E&F).

### UA delayed induction of graft-versus-host disease

**Figure 7:**
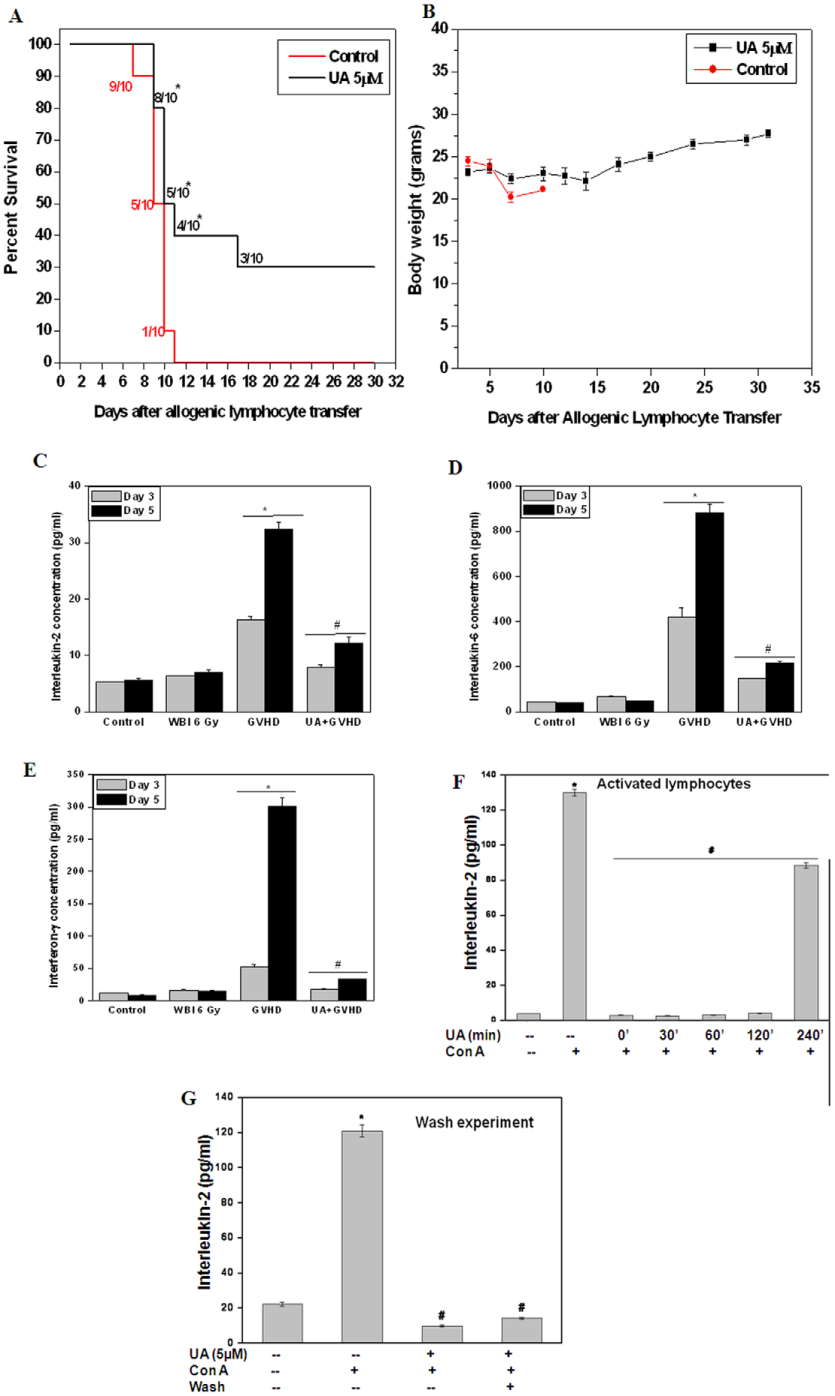
*In vivo* immunosuppressive effect of Ursolic acid. (A, B) Ursolic acid delayed mortality and weight loss in GVHD mice: Eight million lymphocytes from C57BL/6 donor mice were injected i.v. into immunocompromised Balb/c mice 48 h after WBI (600 cGy). Ten mice were included in each group. Control group mice received vehicle treated cells whereas the UA group received cells treated with 5 µM UA for 4 h. (A) Survival of the immunocompromised mice reconstituted with allogenic lymphocytes treated with UA or vehicle. *p<0.05, as compared to mice injected with vehicle treated allogenic lymphocytes. (B) Changes in the body weight of the mice after allo-transplantation. Data points represent mean±SEM from 10 mice. Changes in levels of IL-2 (C), IL-6 (D) and IFN-γ (E) in serum separated from the blood collected on days 3 and 5 from recipient mice (Balb/c) injected with vehicle treated lymphocytes or UA treated lymphocytes isolated from C57BL/6 mice (F) Ursolic acid inhibits cytokine production in activated lymphocytes. Lymphocytes were stimulated with Con A (5 µg/ml) following which UA (5 µM) was added at the indicated time points and the cells were further cultured for 24 h at 37°C. The concentration of IL-2 cytokines in the culture supernatant was estimated using ELISA. (G) Ursolic acid inhibits Con A induced cytokine production even after washing. Lymphocytes were treated with UA (5 µM)) for 4 h and washed with normal RPMI twice, rested for 48 h and then stimulated with Con A (5 µg/ml) for 24 h at 37°C. The concentration of IL-2 in the culture supernatant was estimated using ELISA. Each bar represents mean±S.E.M. from three replicates and two such independent experiments were carried out. *p<0.01, as compared to vehicle treated cells and #p<0.01, as compared to Con A stimulated cells.

To study the in vivo efficacy of UA, we studied its ability to inhibit graft-versus-host disease (GVHD). Splenic lymphocytes from C57BL/6 mice were incubated with UA in vitro (5 µM, 4 h) and adoptively transferred to immunocompromised Balb/c mice. The mice that received untreated control cells developed GVHD that led to 100% death within 10 days (Fig. 7A) and demonstrated typical symptoms of GVHD, including alopecia, scleroderma, hunched posture, diarrhoea, and progressive weight loss. However, in mice that received UA treated cells showed 30% survival in better health for more than 30 days (Fig. 7A). Further, it was observed that mice receiving UA treated lymphocytes experienced inconspicuous weight loss as compared to control group (Fig. 7B). Fig. 7C–E show that on day 3 and day 5 post allo-transplantation, the levels of proinflammatory cytokines (IL-6, IFN-γ and IL-2) were significantly higher in the serum of mice receiving vehicle treated allogenic lymphocytes as compared to those mice which received UA treated allogenic lymphocytes. This observation clearly shows potent anti-inflammatory activity of UA in vivo.

### UA exhibited therapeutic potential by acting post-mitogenic stimulation

To explore its therapeutic potential, UA was added to lymphocytes post-mitogenic stimulation at different time points. It was observed that UA was able to completely suppress Con A induced cytokine secretion in murine lymphocytes even when it was added up to 4 h after mitogenic stimulation (Fig. 7F).

Since UA inhibited the expression of co-stimulatory molecules on activated T cells and B cells, we studied whether UA renders these cells anergic and hence incapable to respond to any further stimuli. We observed that splenic lymphocytes treated with UA for 4 h did not respond to mitogenic stimuli when activated with Con A even though they were washed and rested for 48 h following UA treatment. This shows that UA treated cells are hyporesponsive to antigenic stimulation which might be due to a possible induction of anergy (Fig. 7G).

## Discussion

Triterpenoids form natural components of human diets. An average of 250 mg per day of triterpenes, largely derived from vegetable oils, cereals, fruits and vegetables is consumed [34]. Ursolic acid (UA) is a part of traditional medicine and has been shown to possess many biological activities, such as antioxidative, anti-inflammatory, anticancer and hepato-protective activities [23]. Recent reports indicated that the anticarcinogenic, anti-inflammatory, and proapoptotic effects of ursolic acid were due to its ability to inhibit immunoregulatory transcription factor NFκB in response to a wide variety of carcinogens and inflammatory agents [27]. However, there are no detailed reports on the immunosuppressive effects of UA in T cells which are the primary cell type involved during an adaptive immune response.

To explore the immunomodulatory properties of UA, we studied its effect on T cell activation, proliferation and effector responses to different activating stimuli. We observed that UA was able to inhibit T cell proliferation in response to both polyclonal and antigen specific activation in a dose dependent manner (Fig. 1A–D). These anti-proliferative effects of UA were not due to increased apoptosis in T cells but due to its ability to induce cell cycle arrest in G1 phase (Fig. 1E–G). The immunosuppressive effects of UA were not limited to CD4+ and CD8+ T cells (Fig. 2A–D), but it also suppressed LPS induced proliferation of B cells (Fig. 2E&F) suggesting a common mechanism its of action in these lymphocyte subsets. Cytokines secreted by different cells participating in the immune response are known to play a critical role in successful pathogen clearance. Any alteration in this highly regulated network of cytokines by external or internal factors may result in undesirable consequences. IL-2, TNF-α, and IFN-γ are secreted by Th1 type cells and can activate macrophages and promotes cell-mediated immune responses against invasive intracellular pathogens. Th2 (IL-4, IL-5, IL-6, IL-10, and IL-13) cytokines promote humoral immune responses against extracellular pathogens [35]. UA suppressed both Th1 and Th2 cytokines secreted by activated lymphocytes in response to both polyclonal and antigen specific stimuli in vitro (Fig. 3A–C). Interestingly, UA also mitigated LPS induced secretion of IL-1β, TNF-α and IL-6 in splenic adherent macrophages (Fig. 3D). Quantitative real time RT-PCR for 6 genes which were known to be involved in activation and proliferation of T cells was performed and UA also suppressed the expression of these genes (Fig. 3E). These results suggested that UA acts on several cell types in exhibiting anti-inflammatory activity. Recent investigations suggest cellular redox status may play key role in the regulation of immune responses . Addition of antioxidants has been shown to modulate T cell responses as measured in terms of proliferation and cytokine secretion implicating the importance of ROS in antigen mediated T cell activation [16]. Our group has also recently shown that perturbation of cellular redox status can lead to immunosuppression [33]. Even though UA was observed to increase the basal ROS levels in lymphocytes, the addition of thiol or non-thiol antioxidants could not abrogate the suppressive effects of UA on lymphocyte proliferation and cytokine secretion suggesting a redox independent mechanism of action in lymphocytes (Fig. 5A–D).

Mechanisic studies of anti-inflammatory effects of UA revealed that it acts not only by inhibiting early events in T cell activation but also had potent suppressive effects on the co-stimulatory molecules. It was observed that lymphocytes treated with UA failed to upregulate T cell activation markers CD69, CD25 and CD134 and co-stimulatory marker CD28 upon mitogenic stimulation (Fig. 4A–D). The role of CD28 as the most important co-stimulatory receptor is well established. Engagement of the T cell receptor (TCR) in the absence of CD28 costimulation results in a long-term hyporesponsive state in T cells called clonal anergy and represents one mechanism of peripheral tolerance [36]. Also, the interaction of CD134 with CD134L is known to be involved in T cell activation and also synergize with CD28-B7 co-stimulatory pathway to generate an efficient T cell effector responses [37], [38]. Experiments showing the inhibitory effects of UA on activation and co-stimulatory markers on B cells (CD19, CD80 and CD86) also emphasize its ability to block the co-stimulatory pathway (Fig. 4E–G). The inhibition of LPS induced MHCII on APCs by UA may also aid in its potent anti-inflammatory properties by blocking antigen presentation to T cells (Fig. 4H).

Since many of these lymphocyte activation markers, costimulatory molecules and cytokine genes are under NF-κB and AP-1 regulation, experiments were performed to examine the effect of UA on these transcription factors and MAPKinases. Both ERK and JNK signaling pathways are vital mediators of a number of cellular processes including growth, proliferation, and survival of T cells [39], [40]. We observed that UA was able to inhibit mitogen induced phosphorylation of ERK and JNK and their upstream kinases, MEK and c-raf (Fig. 6A). Further, we also observed that UA was able to inhibit mitogen induced increase in nuclear levels of NF-κB, NF-AT and AP-1 in lymphocytes (Fig. 6B–D). Shishodia et al, have earlier shown suppression of TNF-α induced NF-κB by UA in different tumor cell lines [27]. However, this report shows the effect of UA on mitogen induced NF-κB and its regulated proteins in normal lymphocytes (Fig. 6. B,E&F). Signal 2 mediated by CD28-B7 interaction is required for the induction of NF-κB and AP-1 in Ag-stimulated T cells [41] . This CD28 signaling is provided by the costimulatory molecules B7-1/B7-2 present at the cell surface of APCs [42]. The inhibition of NFκB and AP-1 exhibited by UA might be a consequence of its ability to block the co-stimulatory pathway by down regulating the levels of CD28 and B7. For complete T cell activation and cytokine secretion, the cooperative binding of NF-AT and AP-1 to composite NF-AT/AP-1 binding sites is necessary [43], [44], [45]. The inhibition of NF-AT by UA may lead to inhibition of IL-2 secretion and the observed anti-inflammatory effects. Thus, UA inhibits the activation of the transcription factors NF-AT, NF-κB, and AP-1 which are required to function in a co-ordinated manner to regulate antigen induced immune response.

More importantly, we demonstrated the immunosuppressant activity of UA in vivo. We studied the in vivo anti-inflammatory potential of UA using a mouse model of graft-versus-host-disease which is a frequent complication of allogenic bone marrow transplant in which the engrafted donor T cells attack the recepients' organs and tissues. Clinically, cyclosporine A and tacrolimus have been used in organ transplantation to prevent allograft rejection [46] . However, these drugs are reported to show undesirable side effects that needs to be overcome before they can be used in other inflammatory disorders and autoimmune disease [20], [47]. We observed that treatment of donor lymphocytes with UA prior to allogenic transplantation significantly improved symptoms associated with acute GVHD, delayed GVHD associated mortality and morbidity in recipient mice (Fig. 7A&B) and also decreased the levels of proinflammatory cytokines in the serum (Fig. 7C–E). Interestingly, UA was able to inhibit Con A induced cytokine secretion in murine lymphocytes even when added up to 4 h post-stimulation (Fig. 7F). Consistent with previous results, UA treatment induced a state of hypo-responsiveness in lymphocytes. Further, lymphocytes treated with UA, washed and rested for 48 h showed decreased secretion of IL-2 in response to Con A stimulation (Fig. 7G).

In a recent report by Liu et al., UA induced tolerance to allogenic cardiac transplant in mice was attributed to suppression of NF-κB [48]. They have shown that UA suppressed T cell responses including NF-κB inhibition at 25 µM whereas we have observed that a dose of 5 µM was sufficient to suppress immune cell (T cell, B cell and macrophage) activation. Apart from this, Tao Xu et al., have shown that anti-inflammatory effects of UA are mediated through suppression of transcription regulator RORγt resulting in decreased IL-17 expression in Th17 cells [49]. Further, our studies demonstrate that besides NF-κB suppression UA also suppressed other immunologically important transcription factors AP-1, NF-AT as well as MAPKinases. In conclusion, the present report identified multiple cellular targets of ursolic acid and underlines its application as a potent anti-inflammatory agent with therapeutic potential.

## Materials and Methods

### Chemicals

Ursolic acid, RPMI-1640, HEPES, EDTA, EGTA, PMSF, leupeptin, aprotinin, benzamidine, dithiothreitol (DTT), glutathione (GSH), N-acetyl cysteine (NAC), NP40, propidium iodide, lipopolysaccharide (LPS) and dimethyl sulfoxide (DMSO) were purchased from Sigma Chemical Co. (USA). Fetal calf serum (FCS) was obtained from GIBCO BRL. Concanavalin A (Con A) and trolox were purchased from Calbiochem, USA. ELISA sets for detection of cytokines (IL-2, IL-4, IL-6, IFN-γ, TNF-α and IL-1β) and monoclonal antibodies against Bcl-2, Bcl-xl, CD25, CD69, CD19, CD80, CD86, MHCII, CD134 and CD28 labeled with PE were procured from BD Pharmingen (USA). Antibodies against p-ERK, ERK, IκB-α, p-MEK, p-c-Raf, p-JNK, JNK and β-actin were obtained from Cell Signaling Technologies (USA).

### Approval of animal ethics Committee

‘The Institutional Animal Ethics Committee of Bhabha Atomic Research Centre, Government of India’, has approved the animal studies and the guidelines issued by the ethics committee regarding the maintenance and dissections of small animals were strictly followed. Project No. BAEC/11/10 and Date of approval: April, 2010.

### Treatment with ursolic acid

A 20 mM solution of ursolic acid was prepared in dimethyl sulfoxide, stored as small aliquots at −20°C and diluted as needed in cell culture medium. In all in vitro experiments, cells were treated with different doses of ursolic acid for 4 hours before the initiation of culture. DMSO (0.1%) was used as vehicle control in vitro.

### Proliferation assay

Splenic lymphocytes were obtained by squeezing the spleen through a nylon mesh in a petri plate containing RPMI medium. The RBC were lysed by brief hypotonic shock. Splenic lymphocytes were stained with CFSE (20 µM, 5 min, 37°C) and washed three times using ice-cold RPMI medium containing 10% FCS, 100 IU/ml penicillin and 100 mg/ml streptomycin. Two million splenic lymphocytes were treated with ursolic acid (0.25 µM to 5 µM, 4 h) and were stimulated with Con A (5 µg/ml) or LPS (50 µg/ml) for 72 h at 37°C in 2 ml RPMI with 10% FCS in a 95% air/5% CO_2_ atmosphere. Vehicle treated cells served as a control. Cell proliferation was measured by dye dilution in a flowcytometer (Partec CyFlow). Percent daughter cells that showed a decrease in CFSE fluorescence intensity were calculated using Flowmax® software and were expressed as daughter cells [50].

### CD4+ and CD8+ T cell isolation and proliferation assay

CD4+ (purity: 93%) & CD8+ (purity: 92%) T cells were isolated by using EasySep immunomagnetic cell sorting kit from Stem Cell Technologies, with PE labelled anti-CD4 antibody conjugated to magnetic nanoparticles through dextran and separation using magnetic field. For cell proliferation analysis, total splenocytes were first labelled with CFSE and then sorted and cultured for 24 or 72 h respectively for cytokine or proliferation assay.

### Estimation of cell cycle and apoptosis

The percentage of cells in different phases of cell cycle (G1, S+G2/M) and percentage of apoptotic cells was estimated using a flowcytometer. One million splenic lymphocytes were treated with ursolic acid (0.25–5 µM) for 4 h and stimulated with Con A for 72 h at 37°C in RPMI1640 medium supplemented with 10% FCS in a 5% CO_2_ atmosphere. Vehicle treated cells served as control. The cells were washed with PBS and incubated with 1 ml of staining solution containing 0.5 µg/ml propidium iodide, 0.1% sodium citrate and 0.1% triton X-100 overnight [51]. A total of 20,000 cells were acquired on Partec Cyflow flowcytometer and analyzed using FloMax® software. Undivided cells were in G_1_ phase of cell cycle (2n DNA content). The pre G_1_ population represented the apoptotic cells. The population showing more than 2n DNA represented cells in S+G2/M phase of cell cycle.

### Measurement of cytokine secretion

The concentration of IL-2, IL-4, IL-6 and IFN-γ in the supernatant of control unstimulated cells and cells stimulated with Con A for 24 h after ursolic acid treatment (4 h) was estimated using cytokine ELISA sets (BD Pharmingen, USA). The supernatant obtained from Con A stimulated cells was used as positive control. Cytokines induced by LPS was estimated in the culture supernatant of splenic adherent macrophage. Spleen cells (5×10^6^ cells/well) were incubated in a 24-well cell culture plate for 3 h at 37°C in a humidified atmosphere of 5% CO_2_ and 95% air. The non-adherent cells were removed by aspiration. The adherent cells (macrophages) were incubated with ursolic acid (5 µM for 4 h) and then stimulated with LPS (50 µg/ml) and further cultured for 6 h or 24 h at 37°C. The concentration of IL-6 and TNF-α in the supernatant of LPS stimulated cells for 6 h and IL-1β for 24 h was estimated using cytokine ELISA sets (BD Pharmingen, USA) [52].

**Intracellular ROS measurements:** To detect intracellular ROS, lymphocytes were incubated with 20 µM oxidation-sensitive dichlorofluorescein diacetate (DCF-DA) for 20 min at 37°C before being treated with various concentrations of ursolic acid. After 1 h of incubation, the increase in fluorescence resulting from oxidation of H_2_DCF to DCF was measured using a spectrofluorimeter [53].

### Intracellular GSH assay

To measure intracellular GSH, lymphocytes were treated with ursolic acid for 4 h at 37°C. Monochlorobimane (final concentration, 40 µM, 30 min 37°C) was loaded into cells. Fluorescence emission from cellular sulfhydryl-reacted monochlorobimane was measured using a spectrofluorimeter (BMG Labtech Optima). Monochlorobimane is also known to react with small-molecular-weight thiols other than GSH but GSH forms the major monochlorobimane reactive thiol. Hence, MCB fluorescence is referred to as GSH levels in this manuscript. There are several reports in the literature measuring GSH levels using this dye.

### Intracellular and surface antibody staining

Three million lymphocytes cells were cultured in presence or absence of ursolic acid for 4 h and then stimulated with Con A for 24 h at 37°C. Cultured cells were fixed with 4% paraformaldehyde for 10 min at room temp and excess of paraformaldehyde was removed by washing once with wash buffer (PBS containing 1%BSA). Before staining with monoclonal antibody against Bcl-2 and Bcl-xl, cells were permeabilized with PBST (PBS cotaining0.02% tween-20) thrice for 5 min each at room temperature followed by 2 washes with wash buffer and then incubated with the indicated antibodies for 30 min at room temperature, washed twice and analyzed using a Partec Cyflow flowcytometer.

Surface staining with PE labeled antibodies was done as described earlier [52]. In brief, splenic lymphocytes were treated with ursolic acid (5 µM, 4 h) and were further stimulated with Con A (10 µg/ml) or LPS (50 µg/ml) for 24 h or 48 h. Staining with PE conjugated CD25 antibody or CD69 antibody were done with cells (1×10^6^) obtained after 24 h treatment, while those with CD28, CD134, CD19, CD80, CD86, MHCII were done with cells (1×10^6^) obtained after 48 h treatment. A total 20,000 cells in each group were acquired and analyzed in a Partec Cyflow flowcytometer.

### Western blot analysis

Splenic lymphocytes were treated with ursolic acid (5 µM, 4 h) and were stimulated with Con A (5 µg/ml) for 1 h at 37°C and cytosolic extract or whole cell extract was prepared as described earlier [54] . Vehicle treated cells served as control. Briefly, cells were washed with ice-cold phosphate buffered saline and suspended in 0.1 ml lysis buffer (10 mM HEPES, pH 7.9, 10 mM KCl, 0.1 mM EDTA, 0.1 mM EGTA, 1 mM dithiothreitol, 0.5 mM PMSF, 2 µg/ml leupeptin, 2 µg/ml aprotinin, and 0.5 mg/ml benzamidine). Then cells were allowed to swell on ice for 15 min, after which 25 µl of 10% NP-40 was added and tubes were vortexed. The supernatants containing proteins from cytosolic fraction were collected by centrifuging the cells at 8000 rpm for 6 min at 4°C. The pellet were suspended in nuclear extraction buffer for performing EMSA as described below. Protein estimation was carried out by Bradford method using Bio-Rad Protein Assay Kit. Equal amounts of protein (50 µg) were resolved by SDS-PAGE (10%) and transferred to nitro cellulose membrane. After the membrane was blocked in 5% nonfat powdered milk, it was incubated overnight with the primary antibody specific to IκB-α or p-c-Raf or p-MEK or p-ERK or p-JNK and washed three times with Tris-buffer saline containing 0.05% tween 20 (TBST) and further incubated with horseradish peroxidase-labeled secondary antibody for 1 h. The membranes were washed, and specific bands were visualized on X-ray films using enhanced chemiluminiscence kit (Roche, Germany). The membrane was stripped and reprobed with actin-β or ERK or JNK antibody.

### Electrophoretic mobility shift assay

Splenic lymphocytes were treated with ursolic acid (5 µM, 4 h) and were stimulated with Con A (5 µg/ml) for 1 h at 37°C and nuclear extracts were prepared. The nuclear pellets were resuspended in 25 µl of ice cold nuclear extraction buffer (20 mMHEPES, pH 7.9, 0.4MNaCl, 1 mM EDTA, 1 mMEGTA, 1 mMDTT, 1 mMPMSF, 2.0 pg/ml leupeptin, 2.0 µg/ml aprotinin, and 0.5 mg/ml benzamidine), and the tubes were incubated on ice for 60 min with intermittent agitation. Samples were microcentrifuged for 5 min at 12,000 rpm, and the supernatant was collected in fresh tubes and frozen at −70°C. EMSA was performed by incubating 10 µg of nuclear proteins with 16 fmol of 32 P-end-labeled, double stranded NF-κB oligonucleotides from the human immunodeficiency virus long terminal repeat (5′-TTGTTACAA**GGGACTTTCCGCTGGGGACTTTCC**AGGGAGGCGTGG-3′; boldface indicates NF-κB binding sites) or AP-1(5′-CGCTTGA**TGACTCA**GCCGGAA-3′; boldface indicates AP-1 binding site) or NF-AT(5′-CGC CCA AAG **AGG** AAA ATT TGT TTC ATA-3′; boldface indicates NF-AT binding site) in the presence of 0.5 µg of poly (2′-deoxyinosinic-2′-deoxycytidylic acid) (poly (dI–dC)) in binding buffer (25 mM HEPES, pH 7.9, 0.5 mM EDTA, 0.5 mM dithiothreitol,1% NP 40, 5% glycerol, and 50 mMNaCl) for 30 min at 37°C. The DNA–protein complex formed was separated from free oligonucleotide on 6.6% native polyacrylamide gels using buffer containing 50 mM Tris, 200 mM glycine, and 1 mM EDTA, pH8.5. The dried gel was exposed on phosphorimage plate and the radioactive bands were visualized using a phosphorImage plate scanner (Amersham Biosciences, USA).

### Induction of Graft-Versus-Host Disease (GVHD)

Balb/c mice were exposed to 600 cGy whole body gamma-radiation (WBI) (Gamma Cell 220, AECL Canada). To induce GVHD in immunocompromised Balb/c mice, 8×10^6^ splenic lymphocytes from C57BL/6 donors were injected i.v. 48 h after irradiation. Each mice in control group received vehicle treated splenic lymphocytes, whereas each mice in the ursolic acid group received splenic lymphocytes treated with 5 µM ursolic acid for 4 h. The recipient mice were monitored daily to assess the signs of GVHD. A total of 10 mice were used per group. GVHD became evident from rapid and sustained weight loss as well as from features such as hunchback, diarrhoea, hair loss and death. Serum was separated from the blood collected on days 3 and 5 from recipient mice (Balb/c) injected with vehicle treated lymphocytes or UA treated lymphocytes taken from C57BL/6 mice and levels of different cytokines were estimated using sandwich ELISA.

### Quantitative real-time RT-PCR

mRNA levels in the samples were quantified by quantitative real-time RT-PCR as described previously [55]. Briefly, total RNA was isolated from the samples using Trizol reagent following the manufacturer's instructions (Sigma) and was dissolved in deionised DEPC-treated water. From this RNA 2 µg was converted to cDNA by reverse transcription (cDNA synthesis kit) following the manufacturer's instruction (sigma). qPCR was carried out using the Rotor Gene 3000 (Corbett Research) machine. The PCR was setup by mixing 10× SYBR green PCR mix (sigma) with 5 µl cDNA, 10 picomoles each of forward and reverse primers (Table 1), and PCR-grade water in 20 µl reaction system. The above reaction mixtures were amplified in the following steps: step 1-denaturation at 95°C for 5 min; step 2-denaturation at 95°C for 15 s; step 3-annealing at 57°C for 15 s; step 4-extension at 72°C for 20 s; step 5-melting curve analysis. Steps from 2 to 4 were repeated for 40 cycles. The specificity of respective amplicons was confirmed from the melting curve analysis. The amplification of each gene was carried out in triplicates for each group. The threshold cycle (the cycle at which the amplification enters into exponential phase) values obtained from above runs were used for calculating the expression levels of genes by REST-384 version 2 software [56]. The expressions of genes were normalized against that of a housekeeping gene, β-actin, and plotted as relative change in the expression with respect to control.

**Table 1:** List of primer sequences used for real time quantitative PCR.

Gene	Sequence
**Cdc25a**	Forward: ACAGCAGTCTACAGAGAATGGGReverse: GATGAGGTGAAAGGTGTCTTGG
**E2F**	Forward: CAGAACCTATGGCTAGGGAGTReverse: GATCCAGCCTCCGTTTCACC
**Gadd45g**	Forward: GGGAAAGCACTGCACGAACTReverse: AGCACGCAAAAGGTCACATTG
**Plcg2**	Forward: GTGGACACCCTTCCAGAATATGReverse: ACCTGCCGAGTCTCCATGAT
**Mcm7**	Forward: AGTATGGGACCCAGTTGGTTCReverse: GCATTCTCGCAAATTGAGTCG
**IFN-gamma**	Forward: TGGAGGAACTGGCAAAAGGATGGTReverse: TTGGGACAATCTCTTCCCCAC
**IL-2**	Forward: TGATGGACCTACAGGAGCTCCTGAGReverse: GAGTCAAATCCAGAACATGCCGCAG
**β-actin**	Forward: GCGGGAAATCGTGCGTGACATTReverse: GATGGAGTTGAAGGTAGTTTCGTG

### Statistical analysis

Data are presented as mean ± SEM. The statistical analysis was done using ANOVA with Microcal Origin 6.0 software followed by post-hoc analysis using Schiffe's test. *refers to p<0.01, as compared to vehicle treated control and # refers to p<0.01, as compared to Con A or LPS stimulated cells. Log rank test was used to compare GVHD related mortality in mice injected with control or UA treated allogenic lymphocytes.
